# Supplementary material for: Serotonin N-acetyltransferase SlSNAT2 Positively Regulates Tomato Resistance Against Ralstonia solanacearum
Source: Int J Mol Sci. 2025 Jul 7;26(13):6530. doi: 10.3390/ijms26136530 (PMC12249631; doi:10.3390/ijms26136530)
Supplement: Supplementary file 1 [file ijms-26-06530-s001.zip › ijms-3665156-supplementary.pdf]

Supplemental Information

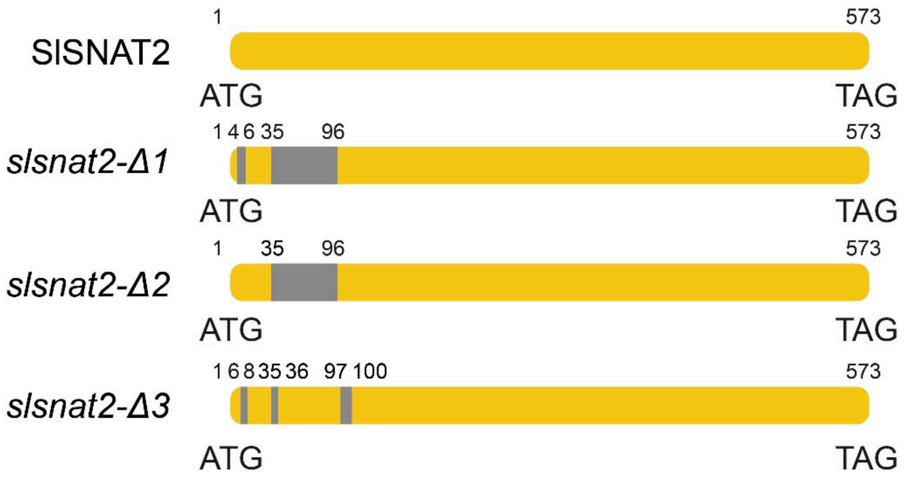

Figure S1 Schematic diagram of SlSNAT2 knockout fragment.

The 'Δ' represents a mutant. The gray area represents the knockout zone. The number represents the base site.

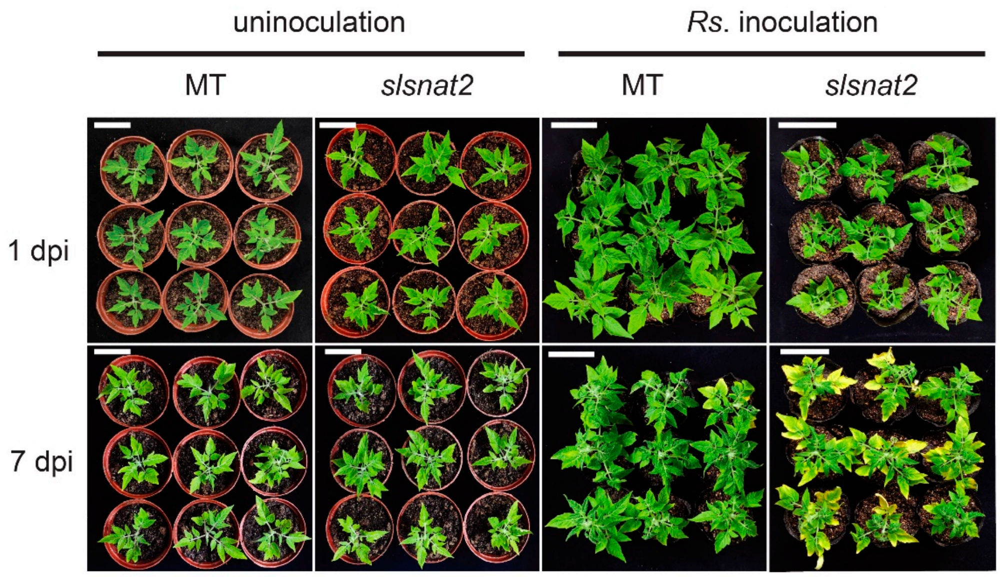

Figure S2 The phenotypes of uninoculated MT and *slsnat2* plants, and MT and *slsnat2* at 1 and 7 dpi with *R. solanacearum*.

The length of the white scale is 5 cm. The *Rs.* stands for *Ralstonia solanacearum*. The *slsant2* contains three various mutant lines, and *slsant2-Δ1*, *slsant2-Δ2* and *slsnat2-Δ3*.

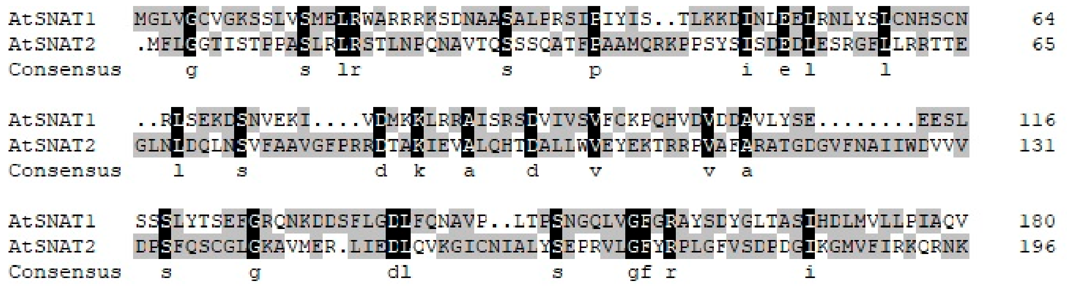

Figure S3 Amino acid sequence homology alignment of AtSNAT1 and AtSNAT2. Numbers represent the count of amino acid.

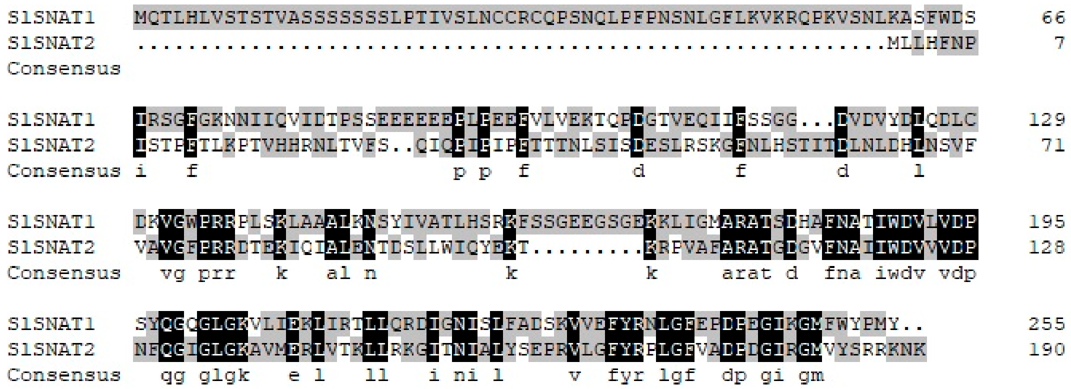

Figure S4 Amino acid sequence homology alignment of SlSNAT1 and SlSNAT2. Numbers represent the count of amino acid.

Table S1 SNAT homologous protein in 13 species. Accession numbers were taken from GenBank, SGN website and NCBI website.

Protein	Species	Accession number
SlSNAT2	*Solanum lycopersicum*	Solyc05g010250
SmSNAT2	*Solanum melongena*	SMEL4_10g010840.1
StSNAT2	*Solanum tuberosum*	KAH0753658.1
NtSNAT2	*Nicotiana tabacum*	XM_016611510.1
CaSNAT2	*Capsicum annuum*	XP_016553285.2
OsSNAT2	*Oryza sativa*	NP_001409299.1
AtSNAT2	*Arabidopsis thaliana*	Q9C666.1
cSNAT	*Synechocystis sp.* PCC 6803	NP_442603

HsSNAT2	*Homo sapiens*	NP_033721.1
MmSNAT	*Mus musculus*	NP_001079
scAANTA	*Saccharomyces cerevisiae*	Q12447.1
FoSNAT	*Fusarium oxysporum* f. sp. *albedinis*	KAJ0142551.1
SpNAT	*Streptococcus pyogenes*	ANC27799.1

Table S2 Primers used in the real-time qRT-PCR analysis of defense related tomato genes. Accession numbers for tomato were taken from GenBank, SGN website.

Pathway	Gene	Accession number	Sequence
MT	*SlSNAT2*	Solyc05g010250	F-CTTCACCCTCAAACCCACC
			R-GAAATCCGACGGCTACAAA
JA	*SlPin2*	AY129402	F-TGATGCCAAGGCTTGTACTAGAGA
			R-AGCGGACTTCCTTCTGAACGT
	SlLoxA	U09026	F-TGGTAGACCACCAACACGAA
			R-GACCAAAACGCTCGTCTCTC
ET	*SlPR-1b*	X14065	F-TTGGTGACTGCGGGATGA
			R-GGCGGCGGCTAGGTT T
	SlOsmotin	M21346	F-TGTACCACGTTTGGAGGACA
			R-ACCAGGGCAAGTAAATGTGC
SA	*SlGluA*	M80604	F-TCA GCA GGG TTG CAA AAT CA
			R-CTCTAGGTGGGTAGGTGTTGGTTAA
	SlPR-1a	M69247	F-GAGGGCAGCCGTGCAA
			R-CACATTTTTCCACCAACACATTG
WRKY	*SlWRKY30*	Solyc10g009550	F-TTTGATCCTCTGGTTCACC
			R-GCTCCATTGCTGCATTTTCT
	SlWRKY81	Solyc09g015770	F-GGACATCACACTTGCAAAGC
			R-AGGCTTCAAATGTTGCTGGA
PR-STHs	*SlPR-STH2a*	Solyc09g0	F-ACATCATGGGTGTCACTAGCT

		90970	R-TACTTCCATCTCCCTCAGCC
	SlPR-STH2b	Solyc09g0<br>90980	F-GCATACCTTCTCGCGAATCC
			R-CAACTTCTACGTCTCTCATTCGA
	SlPR-STH2c	Solyc09g0<br>90990	F-TGTTGAGGGAGATGGTGGTG
			R-TCCATCTCCAGCAGCTTCAA
	SlPR-STH2d	Solyc09g0<br>91000	F-GACTGAGGGAGATGGAAGCA
			R-CAACCTCCATTTCCAGCAGG
MAPK	*SlMPK1*	SGN-<br>U316697	F-CAGATTGTTGCAGGTTTGATC
			R- CAGTCTAAAATAAAATCCACCCCAT
	SlMPK2	SGN-<br>U316695	F- TACTCGCTCGTTTGCTGTTG
			R- TTGGAGTACAGGAAAACAATGG
	SlMPK3	SGN-<br>U313928	F- TGCTAATATGGGTGCTGCTC
			R- TAATTTCACGGAGCGTCCTC
	SlMKK2	SGN-<br>U319074	F- GTTGGATGGCTGCATTTCTC
			R- GAAGATGGAGGAGGCATGG
Actin	*SlACTIN2*	Solyc11g0<br>05330	F-GTCCTCTTCCAGCCATCCAT
			R-ACCACTGAGCACAATGTTACCG
